# Application of a scoring system in Japanese patients diagnosed with atypical hemolytic uremic syndrome to assess the relationship between the score and clinical responses to eculizumab

**DOI:** 10.1186/s12959-023-00489-0

**Published:** 2023-04-18

**Authors:** Hideo Wada, Hirofumi Teranishi, Akihiko Shimono, Noritoshi Kato, Shoichi Maruyama, Masanori Matsumoto

**Affiliations:** 1 Department of General and Laboratory Medicine, Mie Prefectural General Medical Center, Hinaga, Yokkaichi, Mie 5450-132 Japan; 2 Alexion Pharma GK, 3-1-1 Shibaura Minato-Ku, Tokyo, Japan; 3 Department of Nephrology, Nagoya University Graduate School of Medicine, Tsurumai-Cho 65, Showa-ku, Nagoya, Aichi Japan; 4 Department of Blood Transfusion Medicine, Nara Medical University, Shijyo-Cho 840, Kashihara, Nara, Japan

**Keywords:** Atypical hemolytic uremic syndrome, Classification, Diagnosis, Eculizumab, Thrombotic microangiopathy, Post-marketing surveillance

## Abstract

**Background:**

Atypical hemolytic uremic syndrome (aHUS) is caused by complement dysregulation and is generally diagnosed by exclusion from other disorders of thrombotic microangiopathy (TMA). Eculizumab, a terminal complement inhibitor, has been approved for aHUS treatment since 2013 in Japan. Recently, a scoring system was published to support diagnosis of aHUS. Herein we modified this scoring system to apply it to patients diagnosed with aHUS and treated with eculizumab, and assessed the association between the score and clinical responses to eculizumab.

**Methods:**

One hundred eighty-eight Japanese patients who were clinically diagnosed with aHUS, treated with eculizumab, and enrolled in post-marketing surveillance (PMS) were included in this analysis. Some of parameters in the original scoring system were replaced with clinically similar parameters collected in the PMS to modify the system, hereafter referred to as the TMA/aHUS score, which ranges from -15 to 20 points. Treatment responses within 90 days after eculizumab initiation were also assessed, and the relationship between treatment response and TMA/aHUS scores calculated at TMA onset was explored.

**Results:**

The median (range) TMA/aHUS score was 10 (3–16). Receiver operating characteristic curve analysis showed that the cutoff value of TMA/aHUS score to predict treatment response to eculizumab was estimated as 10, and negative predictive value indicated that ≥ 5 points was appropriate to consider assessing the treatment response to eculizumab; 185 (98%) patients had ≥ 5 points and 3 (2%) had < 5 points. Among the patients with ≥ 5 points, 96.1% showed partial response and 31.1% showed complete response. One of the three patients with < 5 points met partial response criteria. No significant difference in the TMA/aHUS scores was observed between survivors and non-survivors, suggesting that the score was not appropriate to predict the outcome (i.e., survival/death) in patients treated with eculizumab.

**Conclusion:**

Almost all patients clinically diagnosed with aHUS scored ≥ 5 points and responded to eculizumab. The TMA/aHUS score system could become a supporting tool for the clinical diagnosis of aHUS and probability of response to treatment with a C5 inhibitor.

**Trial registration:**

This study was conducted as per good PMS practice guidelines for drugs (MHLW Ministerial Ordinance No. 171 of 2004).

**Supplementary Information:**

The online version contains supplementary material available at 10.1186/s12959-023-00489-0.

## Background

Thrombotic microangiopathy (TMA) encompasses a range of severe disorders characterized by hemolytic anemia, thrombocytopenia, and organ damage [1–3]. Thrombotic thrombocytopenic purpura (TTP), typical hemolytic uremic syndrome caused by Shiga toxin-producing Escherichia coli (STEC-HUS), and atypical hemolytic uremic syndrome (aHUS) are clearly recognized as disorders caused by TMA. Other secondary TMAs triggered by various events such as malignant tumors, hematopoietic stem-cell transplantation, and drugs also exist. TTP is due to the deficiency of ADAMTS13, a plasma metalloprotease that cleaves endothelium-derived von Willebrand factor (VWF). STEC-HUS is caused by Shiga toxin-producing *Escherichia coli* (STEC). aHUS, often also referred to as complement-mediated TMA, is caused by the genetic or acquired disposition in the complement alternative pathway resulting in uncontrolled terminal complement activation in association with trigger events; complement-related gene variants cannot be identified in approximately half of aHUS patients, and even patients without complement-related gene variants can have a clear complement dysregulation [4–7]. Due to these different causes, despite clinically being very similar phenotypes, the various TMAs have specific treatment strategies. Owing to its severity, it is vital to differentially diagnose TMAs as quickly as possible to initiate the appropriate therapy in a timely manner [8–11].

Diagnostic criteria for aHUS rely on the exclusion of STEC-HUS, TTP, and secondary TMA because there is no established method to assess the involvement of complement dysregulation in TMA pathophysiology [8–10]. There are reliable clinical tests/markers to exclude STEC-HUS and TTP [12, 13], whereas complete exclusion of the possibility of secondary TMA is often difficult due to lack of established methods, hindering timely and appropriate treatment of aHUS. Even though some biomarkers are expected to estimate complement activation level, there is no reliable and simple clinical marker to accurately diagnose aHUS [14–16]. In addition, even though complement-related gene analysis can be used to confirm the genetic disposition in the complement alternative pathway, this is a lengthy procedure that is unavailable at the point-of-care. Accordingly, diagnosis of aHUS is highly dependent on the physician’s experience and skills.

To support the clinical diagnosis of aHUS, a scoring system was recently published [17]. In the system, two scores are sequentially calculated for TMA screening and aHUS screening respectively. TMA score consists of 4 items ranging 0–10 points, and aHUS score consists of 10 items ranging from -15 to 11 points, in which higher scores indicate higher likelihood of aHUS [17]. To date, no studies have examined the association between the scores and the response to specific treatment for aHUS.

Eculizumab, a recombinant humanized monoclonal anti-C5 antibody, suppresses C5 cleavage by C5 convertase resulting in inhibition of complement overactivation. Eculizumab has been approved for treatment of aHUS in Japan since 2013 [18]. In this study, we used data from Japanese post-marketing surveillance (PMS), which registered pediatric and adult patients who were clinically diagnosed with aHUS and treated with ≥ 1 dose of eculizumab in real-world clinical practice; the design and results of the PMS have been published [19–21].

In this study, the previously published scoring system was modified according to the parameters available in the PMS database, thereby enabling the calculation of TMA/aHUS scores for patients in the database. The association between the score and clinical response to eculizumab treatment was then assessed. Optimum cut-off values to support clinical diagnosis of aHUS were retrospectively estimated using the response criteria.

## Methods

### Study design

PMS is mandated by the Japanese health authorities to monitor the safety and effectiveness of eculizumab in patients clinically diagnosed with aHUS and treated with ≥ 1 dose of eculizumab in real-world settings. The patients with aHUS had been clinically diagnosed in Japan based on the definition in aHUS clinical guide issued in 2013 or updated version in 2016 [9, 10, 22, 23]. The population including patients with aHUS associated with complications such as malignant tumors, transplantations, or autoimmune diseases were also registered in the PMS. They were differentially diagnosed with aHUS (complement-mediated TMA) from secondary TMA. The population analyzed here includes all these patients in the PMS, except for patients whose physicians did not give consent to include their data for publication. The PMS was performed between September 2013 and July 2018. Data locked in August 2021 were used in the current analysis.

Data collected in PMS include patient characteristics, safety, and effectiveness. Patient characteristics include demographics, family history of aHUS, and disease characteristics (genetic mutations or polymorphisms, past treatment, laboratory findings). The decision to initiate treatment with eculizumab was made by the treating physician. Majority of treatment regimens were in accordance with the approved label [18]; however, dosing and administration intervals were determined by the physicians. Follow-up data, including safety and effectiveness, were recorded at 6 and 12 months after starting eculizumab, and annually thereafter [19–21].

### TMA/aHUS scoring system

The scoring system developed by Wada et al. for the diagnosis of aHUS was published previously [17]. The original scoring system was carefully evaluated as a diagnostic scale of aHUS using patients who were diagnosed with aHUS, TTP and secondary TMA and without TMA in the study. Because some parameters used in the original scoring system were not recorded in the PMS, some modifications were applied; Table 1 outlines the components of the modified scoring system, including the definition of ‘trigger’ and ‘underlying disease’. In particular, the item ‘red or brownish urine’, which was not recorded in the PMS was removed, ‘fibrin and degradation product (FDP)’ was replaced with ‘D-dimer’, which was recorded as a coagulation activation marker, and ‘C-reactive protein (CRP)’ was replaced with ‘white blood cell count’, which was recorded as an inflammation activation marker. In addition, some items were defined according to the description of PMS records (see Table 1 legend).

**Table 1:** TMA/aHUS scoring system

Score	Item		Point
TMA score (a)	Hb < 10.0 g/dL^a^		3
	PLT < 150 × 10^9^/L		3
	Renal failure^b^		2
	Extrarenal organ failures^c^		2
	*Possible range*		*0 to 10*
Positive aHUS score (b)^d^	Past history of TMA	1 time	1
		≥ 2 times	3
	Family history of TMA	1 family member	1
		≥ 2 family member	3
	Age at onset	< 63 years	1
		< 10 years	3
	Trigger^e^		1
	*Possible range*		*0 to 10*
Exclusion aHUS score (c)	Bloody stool		− 3
	ADAMTS13 < 10%		− 3
	Underlying diseases^f^		− 3
	D-dimer^g^ > 20 μg/mL		− 3
	WBC^h^ > 16,000/mL		− 3
	*Possible range*		* − 15 to 0*
TMA/aHUS score	= a + b + c		− 15 to 20

^a^Description used in post-marketing surveillance: microangiopathic hemolytic anemia (defined from following items: Hb < 10 g/dL, LDH increase, haptoglobin decrease, presence of schistocyte)

^b^Description used in post-marketing surveillance: acute kidney injury (adults: satisfied diagnostic criteria for acute kidney injury; pediatrics: sCr ≥ 1.5 × age-appropriate ULN)

^c^Two points were given if any of the following applied: neuropsychiatric symptoms, gastrointestinal symptoms, others (thromboembolism, angina pectoris, dyspnea, others) except for fever

^d^Red or brownish urine from the original scoring system was deleted

^e^One point was given for any of the following: viral infection (excluding pneumococcal infection), pregnancy, renal transplantation, or autoimmune and connective tissue diseases

^f^Three points were excluded if any of the following applied: malignant tumor, hematopoietic stem-cell transplantation, chemotherapy acute pancreatitis, or bacterial infection

^g^Modified from fibrin/fibrinogen degradation products (FDP) in the original scoring system

^h^Modified from C-reactive protein (CRP) in the original scoring system

*Abbreviations*: *ADAMTS13* a disintegrin and metalloproteinase with a thrombospondin type 1 motif, member 13, *aHUS* atypical hemolytic uremic syndrome, *Hb* hemoglobin, *PLT* platelet count, *sCr* serum creatinine, *TMA* thrombotic microangiopathy, *ULN* upper limit of normal, *WBC* white blood cell count

In the original scoring system, TMA score and aHUS score were sequentially calculated. In contrast, the current study determined a TMA/aHUS score by scoring thirteen clinically relevant items across three sections (TMA score, positive aHUS score, and exclusion aHUS score), and then calculating a sum of these three sections to simplify the analysis of comparing responses to eculizumab. Items included in the ‘TMA score’ and ‘positive aHUS score’ are assigned positive values, whereas items included in the ‘exclusion aHUS score’ are assigned negative values. The total score ranges from − 15 to 20. Baseline data collected at the time of TMA onset, which was defined by each physician, were used to calculate the baseline TMA/aHUS score. A score value of 5 of this TMA/aHUS score corresponds to the cutoff value to distinguish aHUS from TTP, STEC-HUS and secondary TMA in the original score [17].

### Treatment response assessment

Responses to eculizumab treatment were categorized into partial response and complete response based on changes in hematologic and renal parameters. Partial response was defined as either partial hematologic or renal response, and complete response was defined as both complete hematologic and renal response. Hematologic responses and renal responses were defined to be met if patients met the criteria any time within 90 days from initiation of eculizumab (Table 2). To calculate the TMA/aHUS score after eculizumab treatment to assess the treatment response, clinical data collected at the time point when treatment response criteria were met within 90 days, or otherwise at 90 days after initiation of eculizumab treatment were used.

**Table 2:** Definitions of hematologic and renal responses and Criteria for assessing the treatment response during treatment with eculizumab

**Definitions of hematologic and renal responses**	
Hematologic responses	
Platelet counts	> 100 × 10^9^/L
Hemoglobin	> 10 g/dL
LDH level	< 450 U/L
Renal response	
Improvement of sCr > 25% or dialysis withdrawal	
**Definitions of treatment response criteria**	
Partial response	
Satisfied ≥ one hematologic or renal response criteria	
Complete response	
Satisfied all of the hematologic and renal response criteria	

Each hematologic response or renal response is met if each value is observed any time within 90 days after eculizumab initiation

*Abbreviations*: *aHUS* atypical hemolytic uremic syndrome, *Hb* hemoglobin, *LDH* lactate dehydrogenase, *PLT* platelet count, *sCr* serum creatinine, *TMA* thrombotic microangiopathy

### Statistical analyses

All eligible patients available at the time of the data cut were included in the analyses. Baseline characteristics of patients were analyzed using descriptive statistics. In addition, baseline characteristics of patients were compared among patients stratified by the score of 5, treatment responses (partial response and complete response), and outcomes (death or survival) using Wilcoxon’s rank sum test and Fisher’s test, as appropriate. The cutoff values were estimated using receiver operating characteristic (ROC) curves and negative predictive values calculated from the treatment response and the score value [24]. The score value at baseline and after eculizumab treatment was compared using Wilcoxon’s rank sum test.

## Results

### Patient characteristics enrolled in PMS

Overall, 188 patients in PMS were included in the study. The baseline characteristics of these patients are listed in Supplemental Table 1 (Supplementary material). Of note, Hb was < 10.0 g/dL in 97.3% of patients, PLT count was < 150 × 10^9^/L in 97.9% and renal failure was recorded in 91.0%. Further, 10.1% had a past history of TMA, and 5.6% had a family history of TMA. At the time of TMA onset, 51.1% of the patients were aged ≥ 10 to < 63 years, 26.1% were aged < 10 years, and 22.9% were aged ≥ 63 years. A trigger was noted in 24.5% of patients; the most common trigger was autoimmune disease (12.2%). Regarding the exclusion score items, 16.5% of patients had underlying diseases; malignant tumor was the most common underlying disease (12.2%). The median (range) TMA/aHUS score was 10 (3–16) points.

### Association between clinical response to eculizumab and patient baseline characteristics

Among patients who had post-treatment data in PMS, 174 out of 183 patients (95.1%) met partial response and 56 out of 184 patients (30.4%) met complete response (Table 3). The partial hematologic response, complete hematologic response, and renal response, which were used to define partial/complete response, were achieved in 167/184 (90.8%), 60/184 (32.6%) and 148/183 (80.9%) patients, respectively.

**Table 3:** Baseline characteristics of patients who had post-treatment data recorded in PMS database

	Partial response					Complete response				
	Met		Not met		*p*^†^	Met		Not met		*p*^†^
*N*	174		9			56		128		
**TMA score items**										
Hb < 10.0 g/dL*	169	(97.1)	9	(100)	1.000	55	(98.2)	124	(96.9)	1.000
PLT < 150 × 10^9^/L	171	(98.3)	8	(88.9)	0.184	56	(100)	124	(96.9)	0.315
Renal failure	162	(93.1)	6	(66.7)	0.027	55	(98.2)	113	(88.3)	0.042
Extrarenal organ failures	114	(65.5)	4	(44.4)	0.283	36	(64.3)	83	(64.8)	1.000
**Positive aHUS score items**										
Past history of TMA	16	(9.2)	2	(22.2)	0.217	4	(7.1)	14	(10.9)	0.591
1 time	16	(9.2)	2	(22.2)		4	(7.1)	14	(10.9)	
≥ 2 times	0	(0)	0	(0)		0	(0)	0	(0)	
Family history of TMA	10	(5.7)	0	(0)	1.000	3	(5.4)	7	(5.5)	1.000
1 family member	8	(4.6)	0	(0)		2	(3.6)	6	(4.7)	
≥ 2 family members	2	(1.1)	0	(0)		1	(1.8)	1	(0.8)	
Age at TMA onset										
< 10 years	46	(26.4)	3	(33.3)	0.438	23	(41.1)	26	(20.3)	0.003
≥ 10 to < 63 years	91	(52.3)	3	(33.3)		27	(48.2)	67	(52.3)	
≥ 63 years	37	(21.3)	3	(33.3)		6	(10.7)	35	(27.3)	
Trigger	42	(24.1)	2	(22.2)	1.000	9	(16.1)	35	(27.3)	0.132
**Exclusion aHUS score items**										
Bloody stool	22	(12.6)	0	(0)	0.602	5	(8.9)	17	(13.3)	0.468
ADAMTS13 < 10%	2	(1.1)	0	(0)	1.000	1	(1.8)	1	(0.8)	0.517
Underlying diseases	26	(14.9)	3	(33.3)	0.154	1	(1.8)	28	(21.9)	< 0.001
D-dimer > 20 μg/mL	0	(0)	0	(0)	–	0	(0)	0	(0)	–
WBC > 16,000/mL	2	(1.1)	1	(11.1)	0.141	1	(1.8)	2	(1.6)	1.000
**Total score, median (range)**	10	(3–16)	9	(3–14)	0.155	11	(6–14)	10	(3–16)	< 0.001

Values are *n* (%), unless stated otherwise

^*^Description used in post-marketing surveillance: microangiopathic hemolytic anemia (defined from following items: Hb < 10 g/dL, LDH increase, haptoglobin decrease, presence of schistocyte)

^†^Wilcoxon’s rank sum test and Fisher’s test were used, as appropriate

Abbreviations: *ADAMTS13* a disintegrin and metalloproteinase with a thrombospondin type 1 motif, member 13, *aHUS* atypical hemolytic uremic syndrome, *Hb* hemoglobin, *PLT* platelet count, *sCr* serum creatinine, *TMA* thrombotic microangiopathy, *ULN* upper limit of normal, *WBC* white blood cell count

A significant difference was observed in three parameters (the existence of renal failure, age at TMA onset, and the existence of underlying diseases) between patients who showed partial or complete response and who did not. Patients with partial or complete response were younger (*p* = 0.003 in complete response), and had higher rates of renal failure (*p* = 0.027 and 0.042, respectively) and lower rates of underlying disease (*p* < 0.001 in complete response) at baseline than those without responses (Table 3).

Complement-related genetic variants were found in 45% (47/105) and were not found in 55% (58/105) of patients. No difference of the rate of the presence of genetic variants in the score (*p* = 1.000) or clinical response (partial response: *p* = 1.000, complete response: *p* = 0.684) was observed.

Before the initiation of eculizumab, 56% (104/186) of patients experienced plasma therapy and 48% (90/186) of patients experienced transfusion (Supplemental Table 2, Supplementary material). They might initiate eculizumab due to insufficient response to these therapies, but considering that patients might receive other treatments during eculizumab treatment, the possibility that these treatments affect clinical parameters cannot be excluded.

### Assessment of the cutoff value of the TMA/aHUS score

**Fig. 1:**
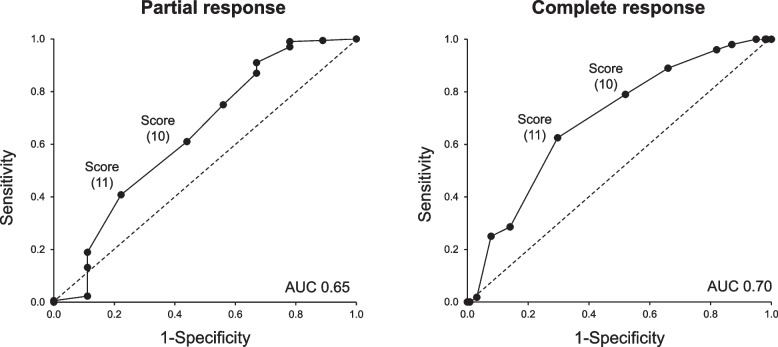
Receiver operating characteristic (ROC) curves for TMA/aHUS scores. Each dot represents ‘Sensitivity’ and ‘1-Specificity’ in each score. Dotted line is a supportive line representing ‘Sensitivity’ = ‘1-Specificity’. Using ROC curve and Youden index analysis, cut-off value can be estimated [25]. Abbreviations: aHUS, atypical hemolytic uremic syndrome; AUC, area under the curve; TMA, thrombotic microangiopathy

We attempted to determine the optimum cutoff value by assessing the relationship between the TMA/aHUS score and the clinical responses to eculizumab. The ROC curves from sensitivity and specificity of treatment response are presented in Fig. 1. At the score of 10 or 11, both sensitivities and specificities were similar values indicating that these points could be potential cutoff values for predicting the response to eculizumab (Table 4). The partial response rate of patients with a score of < 10 or < 11 points were as high as 93.2% (68/73) and 93.6% (103/110), respectively, with similar findings for complete response (supplementary materials, Figures S1 and S2). On the other hand, the odds ratio was highest at a score of 5 for partial response and at a score of 7 for complete response, and negative predictive value (NPV) was highest at a score of 5 for both responses (Table 4). Therefore, the score of 5 could be an optimum cutoff value to consider a clinical response to eculizumab.

**Table 4:** Receiver operating characteristics analysis for each score based on the response for eculizumab treatment

TMA/aHUS score	Partial response					Complete response				
	SE	SP	OR	PPV	NPV	SE	SP	OR	PPV	NPV
≥ 17/ < 17	0.000	1.000	–	–	0.049	0.000	1.000	–	–	0.696
≥ 16/ < 16	0.006	1.000	–	1.000	0.049	0.000	0.992	0.00	0.000	0.694
≥ 15/ < 15	0.006	1.000	–	1.000	0.049	0.000	0.992	0.00	0.000	0.694
≥ 14/ < 14	0.023	0.889	0.19	0.800	0.045	0.018	0.969	0.56	0.200	0.693
≥ 13/ < 13	0.132	0.889	1.22	0.958	0.050	0.250	0.922	3.93	0.583	0.738
≥ 12/ < 12	0.190	0.889	1.87	0.971	0.054	0.286	0.859	2.44	0.471	0.733
≥ 11/ < 11	0.408	0.778	2.41	0.973	0.064	0.625	0.703	3.95	0.479	0.811
≥ 10/ < 10	0.610	0.560	1.95	0.964	0.068	0.790	0.480	3.44	0.400	0.838
≥ 9/ < 9	0.750	0.440	2.36	0.963	0.083	0.890	0.340	4.22	0.370	0.878
≥ 8/ < 8	0.870	0.330	3.45	0.962	0.120	0.960	0.180	5.91	0.340	0.920
≥ 7/ < 7	0.910	0.330	5.30	0.964	0.167	0.980	0.130	**8.42**	0.331	0.944
≥ 6/ < 6	0.970	0.220	9.66	0.960	0.286	1.000	0.050	–	0.316	**1.000**
≥ 5/ < 5	0.990	0.220	**49.43**	0.961	**0.667**	1.000	0.020	–	0.309	**1.000**
≥ 4/ < 4	0.994	0.111	21.63	0.956	0.500	1.000	0.016	–	0.308	**1.000**
≥ 3/ < 3	1.000	0.000	–	0.951	–	1.000	0.000	–	0.304	–

The estimated cutoff value with the highest odds ratio and negative predictive value is indicated in bold

*Abbreviations*: *aHUS* atypical hemolytic uremic syndrome, *SE* sensitivity, *SP* specificity, *NPV* negative predictive value, *OR* odds ratio, *PPV* positive predictive value, *TMA* thrombotic microangiopathy

### Relationship between the proposed cutoff value and treatment response

**Fig. 2:**
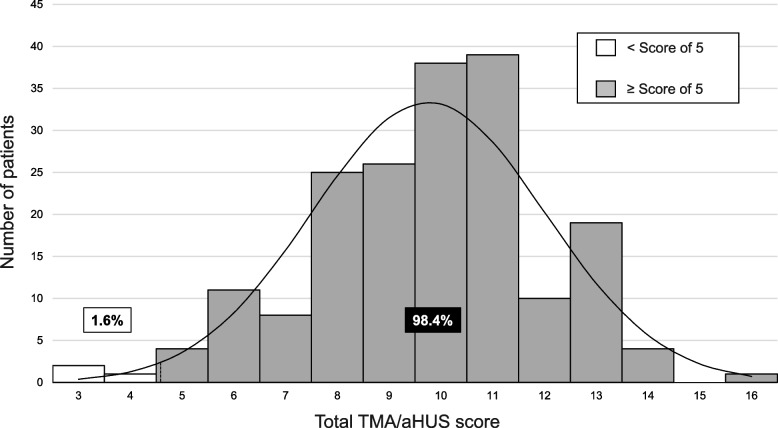
Histogram showing the distribution of patients below and over a cutoff value of 5. White bars represent < cutoff value, and grey bars represent ≥ cutoff value. Solid line indicates the distribution curve; dotted line indicates a lower limit of 2.5%. Abbreviations: aHUS, atypical hemolytic uremic syndrome; TMA, thrombotic microangiopathy

The distribution of score values in each patient is shown in Fig. 2. Among 183 patients with follow-up data, a score of ≥ 5 was observed in 180 patients (98.4%) and < 5 in 3 patients (1.6%). Among 180 patients with a score of ≥ 5, 173 patients (96.1%) showed a partial response and 56 (31.1%) showed a complete response; 7 (3.9%) patients did not show any response (supplementary materials, Figures S1 and S2). Characteristics of patients whose scores were above or below of 5 are also summarized in Supplemental Table 1 (supplementary materials).

### Patients who did not respond to eculizumab

The details of seven patients who did not respond to eculizumab are described in Supplemental Table 3 (supplementary materials). Three patients (#1-#3) died before showing a response to eculizumab, two (#4, #5) had shown improved parameters at the time of eculizumab initiation due to a long period of plasma therapy, one patient’s (#6) platelet count did not recover, and the last patient (#7) discontinued eculizumab treatment before showing responses due to the physician’s decision that “He recovered from a serious condition”.

### Patients with the TMA/aHUS score of < 5 points

The details of 3 patients whose score were < 5 points are described in Supplemental Table 4 (supplementary materials). Two patients (#8, #9) met only one of symptoms of the TMA triad (thrombocytopenia, hemolytic anemia and acute kidney injury) defined in Table 1 at TMA onset. The third patient (#10) was an elderly patient with aHUS following hematopoietic stem-cell transplantation (HSCT). One patient (#8) showed a partial response and the other two patients (#9, #10) did not show any response.

### Treatment outcomes

**Fig. 3:**
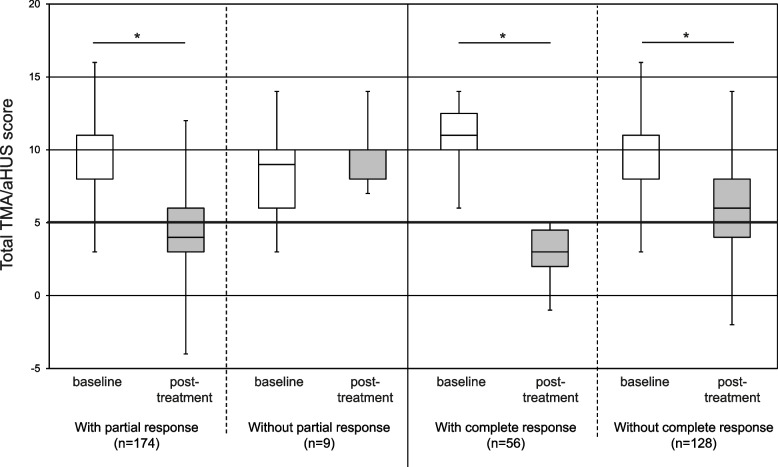
TMA/aHUS score at baseline and after eculizumab treatment. Each box represents the lower (Q1), median (Q2) and the upper (Q3) quartile respectively, and each bar represents data range in each group. Solid horizontal line indicates the diagnostic cutoff value of 5. Changes from baseline were compared using Wilcoxon rank sum test *p < 0.001. Abbreviations: aHUS, atypical hemolytic uremic syndrome; TMA, thrombotic microangiopathy

The TMA/aHUS score of each population before and after eculizumab treatment are shown in Fig. 3. The TMA/aHUS score significantly decreased from baseline after eculizumab treatment (*p* < 0.001) in patients who met any of criteria. The median TMA/aHUS score after treatment decreased to 4 points in patients with partial response and 3 points in patients with complete response.

Overall, 37 (20.0%) patients with the score ≥ 5 died during the observation period. The proportions of patients with an extrarenal organ failure (81.1% vs 62.2%, *p* = 0.033) or an underlying disease (40.5% vs 10.1%, *p* < 0.001) were significantly higher in non-survivors than survivors (Table 5). No significant difference in scores was observed between survivors and non-survivors; the median (range) score was 10 (3–16) in survivors and 9 (4–13) in non-survivors (*p* = 0.055, Table 5). Survivors/non-survivors distribution in each scores is shown in Figure S3 (supplementary materials).

**Table 5:** Outcomes of patients with a TMA/aHUS score ≥ 5 (*N* = 185)

Item	Survivors n (%)	Non-survivors n (%)	*p*^†^
*N*	148 (80)	37 (20)	
**TMA score items**			
Hb < 10.0 g/dL*	145 (98.0)	36 (97.3)	1.000
PLT < 150 × 10^9^/L	146 (98.6)	37 (100.0)	1.000
Renal failure	137 (92.6)	32 (86.5)	0.322
Extrarenal organ failure	92 (62.2)	30 (81.1)	0.033
**Positive aHUS score items**			
Past history of TMA	15 (10.1)	4 (10.8)	1.000
1 time	15 (10.1)	4 (10.8)	
≥ 2 times	0 (0.0)	0 (0.0)	
Family history of TMA, *n*/*N* reported (%)	9/141 (6.1)	1/35 (2.7)	0.689
1 family member	7/141 (4.7)	1/35 (2.7)	
≥ 2 family members	2/141 (1.4)	0/35 (0.0)	
Age at onset of TMA			
< 10 years	37 (25.0)	12 (32.4)	0.673
≥ 10 to < 63 years	77 (52.0)	18 (48.6)	
≥ 63 years	34 (23.0)	7 (18.9)	
Trigger	36 (24.3)	10 (27.0)	0.831
**Exclusion aHUS score items**			
Bloody stool	17 (11.5)	6 (16.2)	0.414
ADAMTS13 < 10%	2 (1.4)	0 (0.0)	1.000
Underlying diseases	15 (10.1)	15 (40.5)	< 0.001
Malignant tumor	11 (7.4)	11 (29.7)	
HSCT	11 (7.4)	10 (27.0)	
Chemotherapy	1 (0.7)	4 (10.8)	
Acute pancreatitis	0 (0.0)	0 (0.0)	
D-dimer > 20 μg/mL	0 (0.0)	0 (0.0)	-
WBC > 16,000/mL	1 (0.7)	1 (2.7)	0.360
**Total score, median (range)**	10 (3–16)	9 (4–13)	0.055

^*^Description used in post-marketing surveillance: microangiopathic hemolytic anemia (defined from following items: Hb < 10 g/dL, LDH increase, haptoglobin decrease, presence of schistocyte)

^†^Wilcoxon’s rank sum test and Fisher’s test were used, as appropriate

*Abbreviations*: *aHUS* atypical hemolytic uremic syndrome, *Hb* hemoglobin, *PLT* platelet count, *sCr* serum creatinine, *TMA* thrombotic microangiopathy, *ULN* upper limit of normal, *WBC* white blood cell count

Twenty-seven of 37 patients who died were reported previously [19–21]. Serious adverse reactions were observed in 12 out of 37 patients. Death due to an adverse reaction was observed in 2 patients; one was reported previously [20] and the other was man in his 50 s who did not have any underlying diseases. He was treated with eculizumab 8 days after TMA onset and died due to extrarenal organ failure 5 days after the second dose of eculizumab.

## Discussion

Here we describe a modified version of an existing aHUS diagnostic scoring system, in which some parameters were replaced with clinically similar parameters collected by PMS’s case report form, and demonstrate the relationships between the TMA/aHUS score and response to eculizumab treatment in clinically diagnosed aHUS patients. Early initiation of treatment with a C5 inhibitor is recommended for patients with aHUS for better outcomes [26], however, currently there are no available/readily usable clinical biomarkers to diagnose aHUS (complement-mediated TMA). The diagnosis of aHUS is highly dependent on the experience of the treating physicians, which can post challenges to initiate treatment with a C5 inhibitor in a timely manner. The TMA/aHUS scoring system presented here could be utilized as a supportive tool for aHUS diagnosis, and in patients with a score of ≥ 5, initiation of treatment of eculizumab could be considered.

Cut-off values of the modified scoring system were independently calculated and assessed using ROC curve analysis and negative predictive values in relation to treatment response to eculizumab, which could indicate complement-mediated TMA in patients. Cut-off value calculated from ROC curves showed high scores (10 or 11 points), likely because all patients in PMS were clinically diagnosed with aHUS. In contrast, the cut-off value derived from negative predictive values (5 points) could be clinically more appropriate to not miss patients who show any responses to C5 inhibitor, even if false positive might be concerned.

The coincidence in the cut-off value of 5 points independently calculated from original and modified scoring systems might suggest a similarity of those systems in differential diagnosis of aHUS. Unfortunately, this study utilized only the patients clinically diagnosed with aHUS and did not address differences of the scores in patients with aHUS, secondary TMA and other TMAs. In contrast, the original scoring system illustrated the importance of positive aHUS score and exclusion aHUS score for differential diagnosis of aHUS from other TMAs; the sum of positive and exclusion scores (range) was 5 (2–6) for aHUS, -3 (-5 to -2) for TTP, -3 (-5 to -2) for secondary TMA and -2.5 (-5 to 0) for TMA without aHUS. Thus, the original scoring system was carefully evaluated using patients diagnosed with aHUS, TTP, and secondary TMA, and volunteers without TMA, but the modified system has not been applied to patients without aHUS; therefore, a validation of the modified system will be required for further application. Importantly, 98% of patients clinically diagnosed with aHUS in PMS showed a TMA/aHUS score ≥ 5, which corresponded to the cut-off value in the original diagnostic score system [17]; moreover, 96% showed treatment response to eculizumab, which might be evidence of complement-mediated TMA. These data suggest a relationship between a TMA/aHUS score ≥ 5 and a response to eculizumab, which in combination with physician expertise may help determine the proper treatment for each patient.

In addition, this study also did not address the differences of clinical responses in patients with aHUS (with trigger or underlying disease), secondary TMA and other TMAs. Therefore, further study will be needed to see the relationship between the score and clinical response to supportive care, PE/PI, or eculizumab in various TMAs.

In the current analysis, we applied early response criteria to patients enrolled in the PMS: patients were defined as responders if hematologic or renal parameters improved at any time within 90 days. When patients do not respond to C5 inhibitor, other treatment options for possible causes of TMA should be considered [8–10, 22, 23]. Among the patients who met a TMA/aHUS score ≥ 5, seven patients did not demonstrate a response to eculizumab treatment. In contrast, there was one patient who showed renal function improvement after the initiation of eculizumab treatment despite the fact that his TMA/aHUS score was < 5 points. Therefore, in both patients who meet and do not meet the cut-off value, clinical signs of TMA and response to treatment should be carefully monitored after the initiation of eculizumab. The current analysis indicates that the TMA/aHUS score significantly decreases from baseline in patients who meet treatment response. This scoring system could thereby also be used as a supportive tool to evaluate eculizumab treatment response.

Both the original and modified scoring systems included the positive aHUS scored items “prodromal symptom” or “trigger”, defined as conditions which can induce complement overactivation causing aHUS; on the other hand, the exclusion aHUS score parameters included “underlying disease”. Interestingly, we demonstrated that 30/31 patients with underlying disease were scored ≥ 5, and that many of them showed partial response to eculizumab, indicating that these patients can be treated with eculizumab after appropriate clinical diagnosis with aHUS. However, the underlying diseases including malignant tumor, HSCT and chemotherapy may affect clinical characteristics in addition to TMA, making fully recovery difficult only with treatment of TMA. That might be why patients who did not show complete response had higher rate of underlying diseases.

In addition, patients who showed treatment response were younger and had higher rate of renal failure at the onset of TMA. The younger age of TMA onset might be correlated with renal dysfunction, since younger age of TMA onset predicted the risk of end-stage-renal-disease [adjusted hazard ratio 0.55 (95% confidence interval 0.41–0.73)] in a natural history study from the Global aHUS registry [6].

Considering that significant difference was not observed in the median score between survivors and non-survivors, the score value and the outcome (i.e., survival/death) seem to be independent, indicating that eculizumab treatment should not be delayed if the score value is ≥ 5. It is important to note that the proportions of patients with extrarenal organ failure and underlying diseases were significantly higher among non-survivors. This result is consistent with that of a long-term safety analysis of 1321 eculizumab-treated patients with aHUS, in which 58 deaths were reported [27]; in this analysis, the most common causes of death were infection, cancer, and cardiovascular events. These findings demonstrate the importance of managing any underlying diseases/infections and organ failure to improve the prognosis of patients with aHUS.

In the present study, patients who were clinically diagnosed with aHUS and received eculizumab were analyzed. Therefore, we were not able to examine the scores and response to eculizumab in patients without aHUS, or with aHUS who did not receive eculizumab. Additional studies targeting a wider range of patients other than the PMS are thereby needed to properly validate the aHUS diagnostic cutoff score of 5 using the TMA/aHUS score, and further assess its utility to predict treatment response with C5 inhibitors.

## Conclusions

This analysis of Japanese eculizumab PMS data using the TMA/aHUS score confirms that scoring systems could become a supportive tool for diagnosis of aHUS and could be further developed as a potential method to predict treatment response.

## Supplementary Information

**Additional file 1:** **Table S1.**Overall patient characteristics at baseline. **Table S2.** Plasma therapy and transfusion before the treatment with eculizumab. **Table S3.** Patients who did not respond to eculizumab. **Table S4.**Patients with the TMA/aHUS score of <5 points. **Figure S1.** Relationship between TMA/aHUS score cutoff values and response to eculizumab based on partial response. **Figure S2.** Relationship between TMA/aHUS score cutoff values and response to eculizumab based on complete response. **Figure S3.** Histogram showing the distribution of survivors and non-survivors during observation period in each score.

## Data Availability

The datasets generated and analyzed during the current study are not publicly available because data cannot be shared to protect personal information.

## Abbreviations

aHUS: Atypical hemolytic uremic syndrome
TMA: Thrombotic microangiopathy
PMS: Post-marketing surveillance
TTP: Thrombotic thrombocytopenic purpura
STEC-HUS: Shiga toxin-producing Escherichia coli
PLT: Platelet
Hb: Hemoglobin
LDH: Lactate dehydrogenase
ROC: Receiver operating characteristic
PPV: Positive predictive value
NPV: Negative predictive value
sCr: Serum creatinine
ADAMTS13: A disintegrin and metalloproteinase with a thrombospondin type 1 motif, member 13
ULN: Upper limit of normal
WBC: White blood cell count
