# Supplementary material for: Application of a scoring system in Japanese patients diagnosed with atypical hemolytic uremic syndrome to assess the relationship between the score and clinical responses to eculizumab
Source: Thromb J. 2023 Apr 18;21:43. doi: 10.1186/s12959-023-00489-0 (PMC10114489; doi:10.1186/s12959-023-00489-0)

**Supplemental TABLE 1.** Overall patient characteristics at baseline

| **Item** | **Overall, *n* (%)** | **TMA/aHUS score** | | |
| --- | --- | --- | --- | --- |
|  |  | **≥5, *n* (%)** | **<5, *n* (%)** | ***p***^†^ |
| *N* | 188 | 185 | 3 |  |
| TMA score items |  |  |  |  |
| Hb <10.0 g/dL* | 183 (97.3) | 181 (97.8) | 2 (66.7) | 0.078 |
| PLT <150 × 10^9^/L | 184 (97.9) | 183 (98.9) | 1 (33.3) | 0.001 |
| Renal failure | 171 (91.0) | 169 (91.4) | 2 (66.7) | 0.248 |
| Extrarenal organ failures | 123 (65.4) | 122 (65.9) | 1 (33.3) | 0.274 |
| Positive aHUS score items |  |  |  |  |
| Past history of TMA | 19 (10.1) | 19 (10.3) | 0 (0.0) | 1.000 |
| 1 time | 19 (10.1) | 19 (10.3) | 0 (0.0) |  |
| ≥2 times | 0 (0.0) | 0 (0.0) | 0 (0.0) |  |
| Family history of TMA, *n*/*N* reported (%) | 10/179 (5.6) | 10 (5.4) | 0 (0.0) | 1.000 |
| 1 family member | 8/179 (4.5) | 8 (4.3) | 0 (0.0) |  |
| ≥2 family members | 2/179 (1.1) | 2 (1.1) | 0 (0.0) |  |
| Age at onset of TMA |  |  |  | 0.194 |
| <10 years | 49 (26.1) | 49 (26.5) | 0 (0.0) |  |
| ≥10 to <63 years | 96 (51.1) | 95 (51.4) | 1 (33.3) |  |
| ≥63 years | 43 (22.9) | 41 (22.2) | 2 (66.7) |  |
| Trigger | 46 (24.5) | 46 (24.9) | 0 (0.0) | 1.000 |
| Infections (exclude pneumococcal infection) | 14 (7.4) | 14 (7.6) | 0 (0.0) |  |
| Pregnancy | 1 (0.5) | 1 (0.5) | 0 (0.0) |  |
| Renal transplantation | 12 (6.4) | 12 (6.5) | 0 (0.0) |  |
| Autoimmune disease | 23 (12.2) | 23 (12.4) | 0 (0.0) |  |
| Exclusion aHUS score items |  |  |  |  |
| Bloody stool | 23 (12.2) | 23 (12.4) | 0 (0.0) | 1.000 |
| ADAMTS13 <10% | 2 (1.1) | 2 (1.1) | 0 (0.0) | 1.000 |
| Underlying diseases | 31 (16.5) | 30 (16.2) | 1 (33.3) | 0.419 |
| Malignant tumor | 23 (12.2) | 23 (12.4) | 0 (0.0) |  |
| HSCT | 22 (11.7) | 21 (11.4) | 1 (33.3) |  |
| Chemotherapy | 5 (2.7) | 5 (2.7) | 0 (0.0) |  |
| Acute pancreatitis | 0 (0.0) | 0 (0.0) | 0 (0.0) |  |
| D-dimer >20 μg/mL | 0 (0.0) | 0 (0.0) | 0 (0.0) | - |
| WBC >16,000/mL | 3 (1.6) | 2 (1.1) | 1 (33.3) | 0.047 |
| Total score, median (range) | 10 (3–16) |  |  |  |

*Description used in post-marketing surveillance: microangiopathic hemolytic anemia (defined from following items: Hb <10 g/dL, LDH increase, haptoglobin decrease, presence of schistocyte).

^†^Wilcoxon’s rank sum test and Fisher’s test were used, as appropriate.

Abbreviations: ADAMTS13, a disintegrin and metalloproteinase with a thrombospondin type 1 motif, member 13; aHUS, atypical hemolytic uremic syndrome; Hb, hemoglobin; PLT, platelet count; sCr, serum creatinine; TMA, thrombotic microangiopathy; ULN, upper limit of normal; WBC, white blood cell count

**Supplemental TABLE 2.** Plasma therapy and transfusion before the treatment with eculizumab

|  | Total | Adults (≥18 years old) | Pediatrics (<18 years old) |
| --- | --- | --- | --- |
| Number of patients, N | 186 | 121 | 65 |
| Plasma therapy, N (%) | 104 (56) | 69 (57) | 35 (54) |
| Transfusion, N (%) | 90 (48) | 56 (46) | 34 (52) |

**Supplemental TABLE 3.** Patients who did not respond to eculizumab

| Patient No. | Sex | Age at TMA onset, y | Score | Days from TMA onset to ECZ treatment | ECZ duration | Outcome | Cause of death | Other details |
| --- | --- | --- | --- | --- | --- | --- | --- | --- |
| 1 | F | 2 | 8 | 6 | 7 | dead | multi organ dysfunction syndrome | She received hematopoietic stem cell transplantation before TMA onset. She died 24 days after eculizumab initiation. This case was reported in the previous report [19]. |
| 2 | F | 80s | 6 | 16 | 15 | dead | liver dysfunction | She did not have triggers nor underlying diseases. She died after 22 days after eculizumab initiation. This case was reported in the previous report [20]. |
| 3 | F | 60s | 10 | 4 | 1 | dead | gastrointestinal bleeding | She received renal transplantation before TMA onset. She died 9 days after eculizumab initiation. |
| 4 | M | 40s | 10 | 277 | 2073 | alive | - | Before eculizumab treatment, he received plasma therapy. Both hematologic and renal marker became normal until eculizumab initiation. |
| 5 | M | 7 | 14 | 546 | 1674 | alive | - | Before eculizumab treatment, he received plasma therapy. Hematologic marker became normal until eculizumab initiation, but renal dysfunction remained to be observed until 90 days after eculizumab initiation. He received dialysis throughout the observation period. |
| 6 | M | 40s | 9 | 78 | 39 | alive | - | His LDH level and renal function was normal at eculizumab initiation, and during 90 days after eculizumab initiation. His platelet count was low (66 x10^9^/L) at TMA onset and did not recover. Eculizumab treatment was discontinued due to insufficient response. |
| 7 | M | 2 | 11 | 8 | 22 | alive | - | He received hematopoietic stem cell transplantation before TMA onset. He discontinued eculizumab treatment by the physician’s decision as "He got out of a serious condition" before hematologic and renal response were observed. |

Abbreviations: aHUS, atypical hemolytic uremic syndrome; TMA, thrombotic microangiopathy; ECZ, eculizumab

**Supplemental TABLE 4.** Patients with the TMA/aHUS score of <5 points

| Patient No. | Sex | Age at TMA onset, y | Score | Days from TMA onset to ECZ treatment | ECZ duration | Response | Outcome | Cause of death | Other details |
| --- | --- | --- | --- | --- | --- | --- | --- | --- | --- |
| 8 | M | 40s | 3 | 294 | 57 | partial | alive | - | Eculizumab was initiated by the physician's decision from the result that renal tissue was positive for C5a and C5b-9 staining. Hematologic markers were normal throughout the observation period and only renal impairment was observed. His renal function improved during eculizumab treatment. |
| 9 | M | 70s | 3 | 66 | 174 | none | alive | - | He was clinically diagnosed with aHUS based on microangiopathic hemolytic anemia (high LDH level) and schistocytes, and the exclusion of TTP and STEC-HUS. Neither platelet decrease nor renal dysfunction were observed at TMA onset. At the time of eculizumab initiation, renal dysfunction was observed. |
| 10 | F | 60s | 4 | 9 | 8 | none | dead | GVHD, multiple organ dysfunction syndrome | She was clinically diagnosed with aHUS due to TMA symptoms, and the exclusion of TTP and STEC-HUS. She met two exclusion aHUS score items: history of hematopoietic stem-cell transplantation and high white blood cell count. This case was reported in the previously [20]. |

Abbreviations: aHUS, atypical hemolytic uremic syndrome; TMA, thrombotic microangiopathy; GVHD, graft-versus-host disease; ECZ, eculizumab

**FIGURE S1.** Relationship between TMA/aHUS score cutoff values and response to eculizumab based on partial response.

Values are shown as the percentage of patients with/without a response (overall and according to the specified cutoff score).

For each cutoff score, the overall percentage of patients ≥cutoff value (blue) and <cutoff value (red) is indicated, along with the percentages meeting (light yellow) or not meeting (white) partial response within each subgroup.

Abbreviations: aHUS, atypical hemolytic uremic syndrome; TMA, thrombotic microangiopathy

**
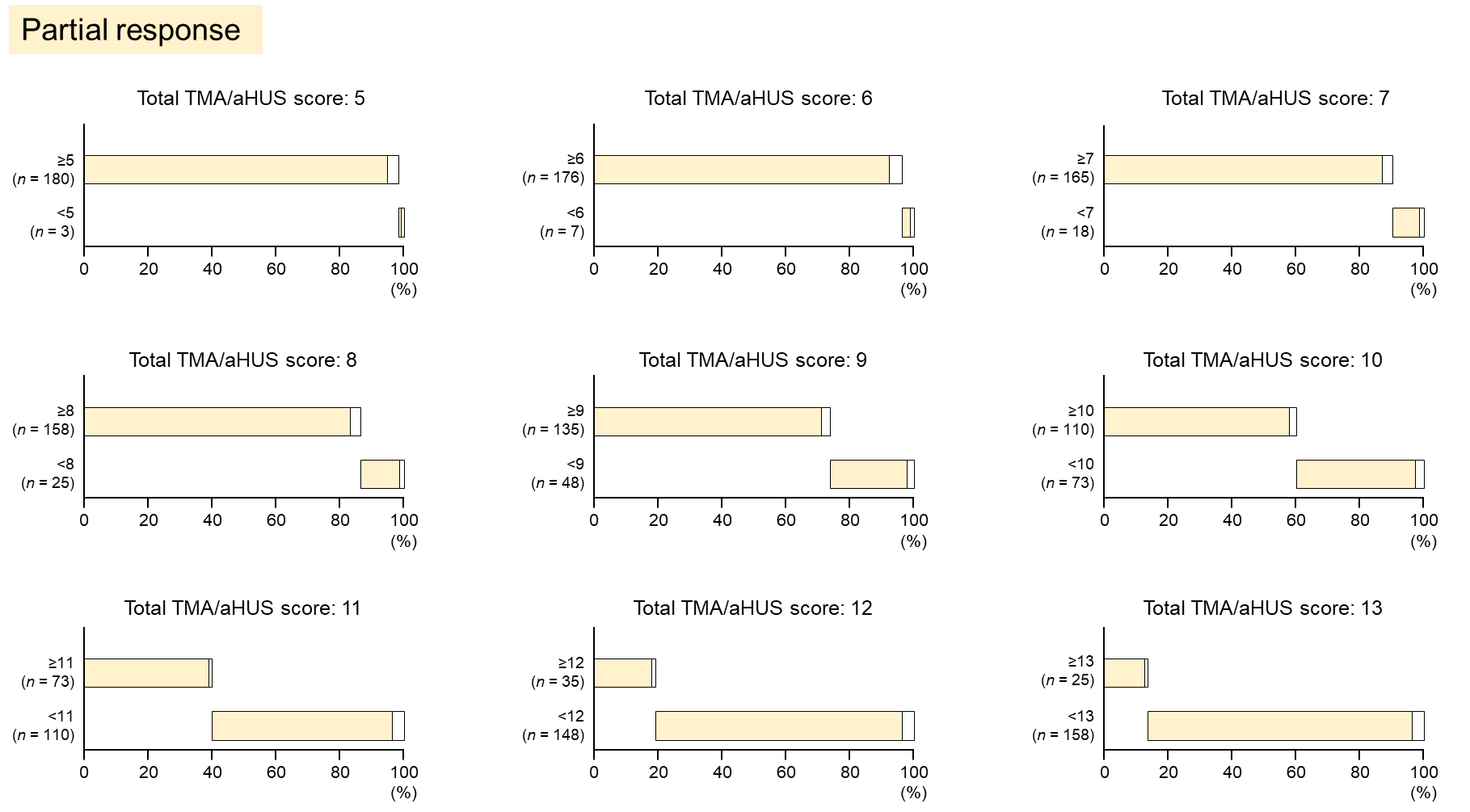
**

**FIGURE S2.** Relationship between TMA/aHUS score cutoff values and response to eculizumab based on Complete response

Values are shown as the percentage of patients with/without a response (overall and according to the specified cutoff score).

For each cutoff score, the overall percentage of patients ≥cutoff value (blue) and <cutoff value (red) is indicated, along with the percentages meeting (dark yellow) or not meeting (white) Complete response within each subgroup.

Abbreviations: aHUS, atypical hemolytic uremic syndrome; TMA, thrombotic microangiopathy

**
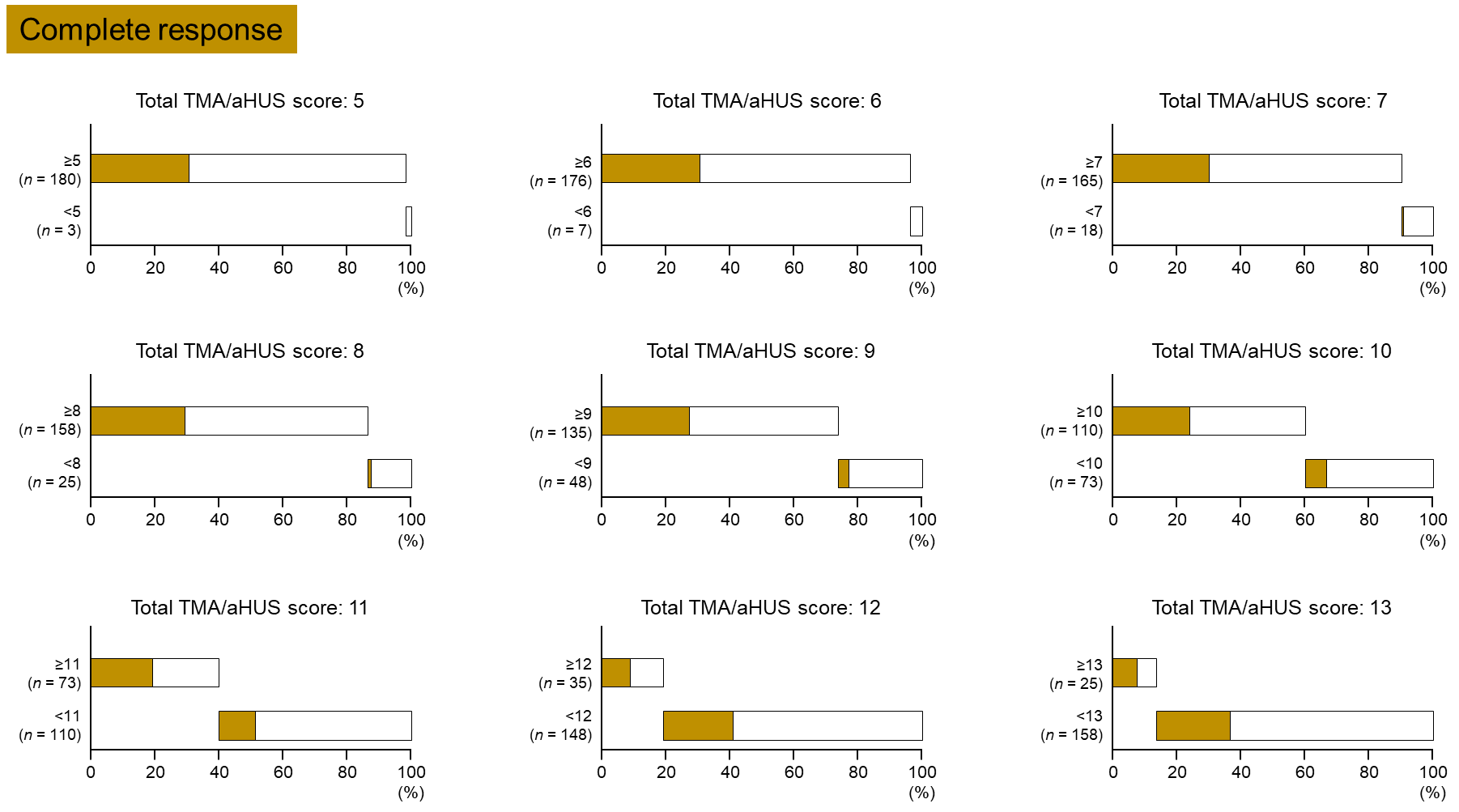
**

**FIGURE S3.** Histogram showing the distribution of survivors and non-survivors during observation period in each score. Survivor are shown in white and non-survivors are shown in grey.

Abbreviations: aHUS, atypical hemolytic uremic syndrome; TMA, thrombotic microangiopathy


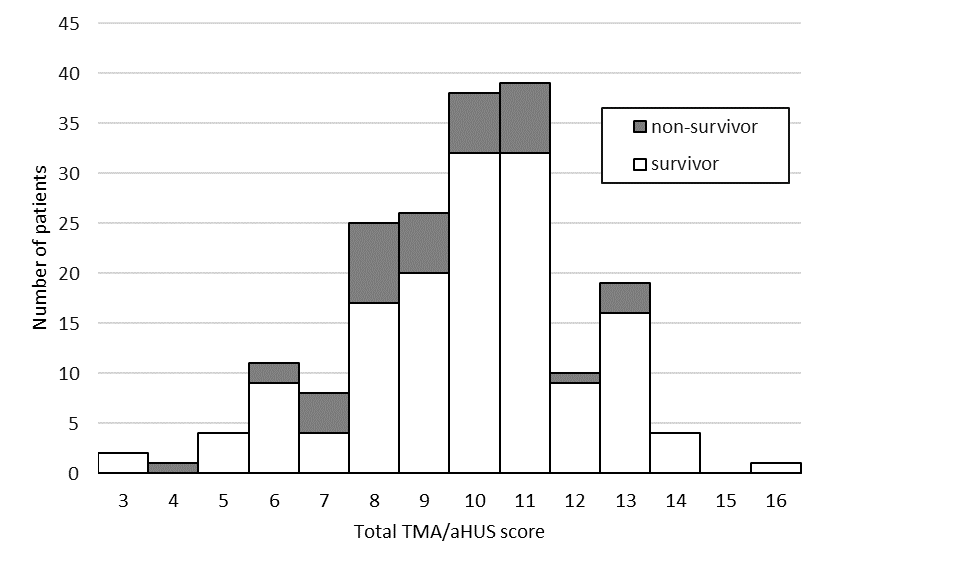

Supplement: Supplementary file 1 — Additional file 1: Table S1.Overall patient characteristics at baseline. Table S2. Plasma therapy and transfusion before the treatment with eculizumab. Table S3. Patients who did not respond to eculizumab. Table S4.Patients with the TMA/aHUS score of <5 points. Figure S1. Relationship between TMA/aHUS score cutoff values and response to eculizumab based on partial response. Figure S2. Relationship between TMA/aHUS score cutoff values and response to eculizumab based on complete response. Figure S3. Histogram showing the distribution of survivors and non-survivors during observation period in each score. [file 12959_2023_489_MOESM1_ESM.docx]
